# The role of local renin-angiotensin system in arterial chemoreceptors in sleep-breathing disorders

**DOI:** 10.3389/fphys.2014.00336

**Published:** 2014-09-05

**Authors:** Man Lung Fung

**Affiliations:** Department of Physiology, University of Hong KongPokfulam, Hong Kong

**Keywords:** angiotensin II, AT_1_ receptor, carotid body, intermittent hypoxia, OSA

## Abstract

The renin-angiotensin system (RAS) plays pivotal roles in the regulation of cardiovascular and renal functions to maintain the fluid and electrolyte homeostasis. Experimental studies have demonstrated a locally expressed RAS in the carotid body, which is functional significant in the effect of angiotensin peptides on the regulation of the activity of peripheral chemoreceptors and the chemoreflex. The physiological and pathophysiological implications of the RAS in the carotid body have been proposed upon recent studies showing a significant upregulation of the RAS expression under hypoxic conditions relevant to altitude acclimation and sleep apnea and also in animal model of heart failure. Specifically, the increased expression of angiotensinogen, angiotensin-converting enzyme and angiotensin AT_1_ receptors plays significant roles in the augmented carotid chemoreceptor activity and inflammation of the carotid body. This review aims to summarize these results with highlights on the pathophysiological function of the RAS under hypoxic conditions. It is concluded that the maladaptive changes of the RAS in the carotid body plays a pathogenic role in sleep apnea and heart failure, which could potentially be a therapeutic target for the treatment of the pathophysiological consequence of sleep apnea.

## Introduction

**Figure 1:**
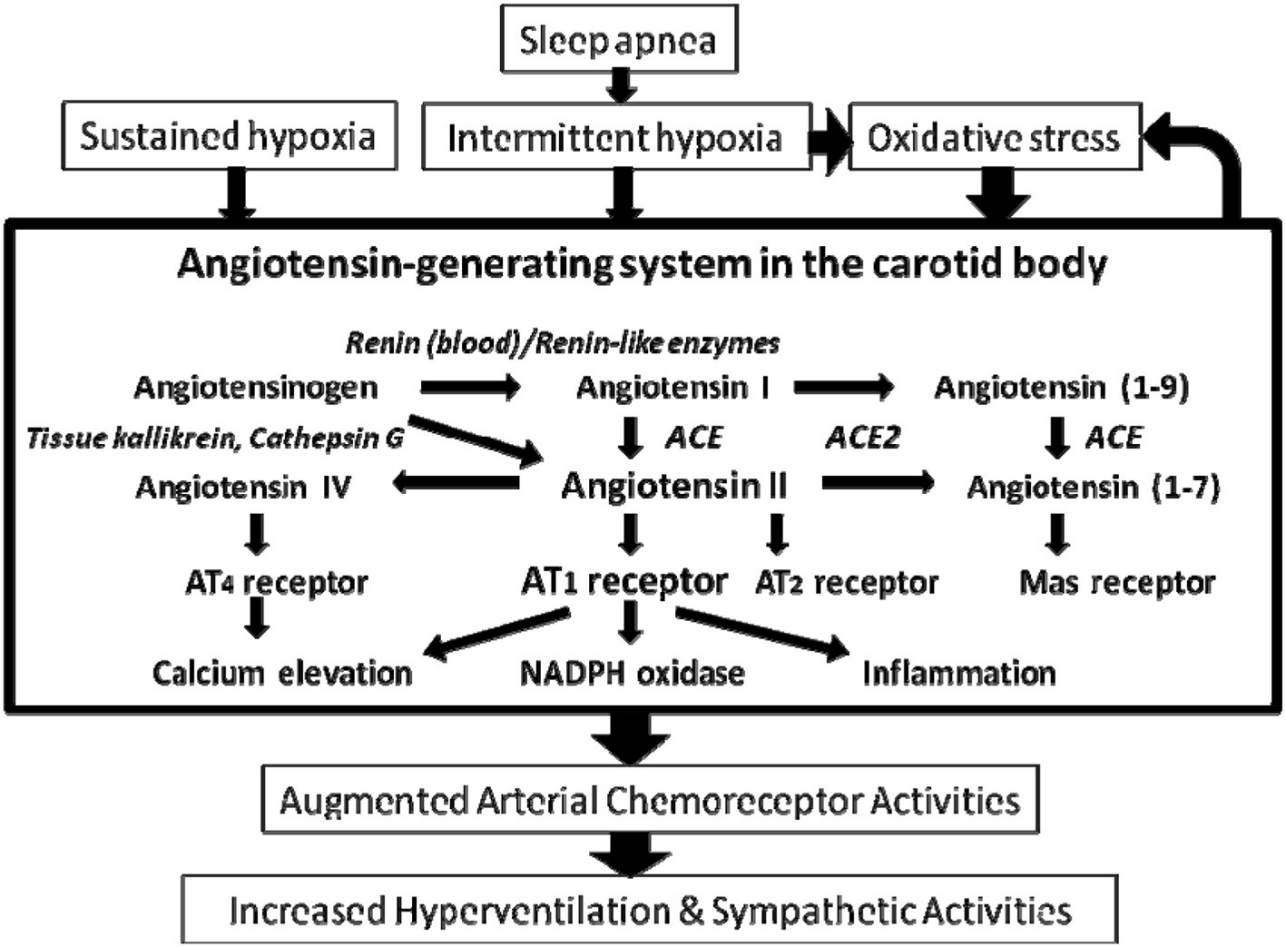
**Main components of the renin angiotensin system and the enzymes mediating the proteolytic process in the carotid body**. Arrows denote the main direction of the physiological or pathophysiological cascade. Note the renin-independent biosynthetic pathway and the enzymes involved are shown in italic. The major physiological effect of Ang II is mediated by the AT_1_ and AT_2_ receptors. In addition, the Ang II metabolites Ang IV and Ang (1-7) exert biological effects via AT_4_ and Mas receptors, respectively. ACE, angiotensin-converting enzyme.

The renin-angiotensin system (RAS) plays important physiological roles in the humoral regulation of blood pressure, electrolyte and fluid homeostasis (Peach, 1977). The physiological effects are mediated by bioactive angiotensin (Ang) peptides including Ang II, Ang III, Ang IV, and Ang (1-7), produced by renin, angiotensin-converting enzyme (ACE), ACE-2 and angiotensin-processing peptidases, via the angiotensin AT_1_, AT_2_, AT_4_, and Mas receptors (Figure 1). In addition to the endocrine function of RAS, growing amount of evidence demonstrates that the intrinsic RAS functions in an autocrine-paracrine manner, mediated by the RAS components expressed in numerous tissues and organs. The local RAS has diverse effects on, for examples, the regulation of vascular tone, generation of reactive oxygen species (ROS), inflammation or fibrogenesis, which are regulated by various physiological stimuli or pathophysiological conditions (Campbell, 2003). As such, it has been proposed that the local RAS might be a potential therapeutic target for the treatment of disease.

Arterial chemoreceptors in the carotid body are important for the rapid adjustment of respiratory and cardiovascular activities via the chemoreflex elicited by the sensory afferent activity of the chemoreceptor responding to changes in chemical stimuli in the arterial blood. The carotid body is a highly vascularized organ with blood perfusion far exceeding the needs of its local tissue metabolism. Thus, changes in arterial oxygen tension or pH, circulating humoral and locally produced signaling substances acting as paracrines or autocrines can readily diffuse to the chemosensory components of the carotid body. In addition to the response to hypoxia, hypercapnia and acidosis, the carotid chemoreceptor responds to Ang II because AT receptors are expressed in the chemosensitive glomus cell of the carotid body (Allen, 1998; Fung et al., 2001). Moreover, the level of RAS gene expression in the carotid body is regulated by hypoxia (Leung et al., 2000; Fung et al., 2002). In this context, the augmented activity of the carotid body has been proposed to play a role in the pathophysiology of sleep apnea. Hence, the molecular and cellular mechanisms that mediate the effect of hypoxia on the RAS in the carotid body and the pathophysiological role of the RAS in disease conditions associated with hypoxemia are of great interest. Research studies have been focused on: (a) mechanisms underlying the carotid chemoreceptor response to Ang II; (b) the expression of RAS components in the carotid body; (c) the regulation of the RAS expression in the carotid body, and (d) pathophysiological roles of the alteration of the RAS in the carotid body in disease conditions associated with hypoxemia. This review aims to summarize the literature on the functional expression of RAS in the carotid body and its regulation by hypoxia, highlighting the pathophysiological roles of the RAS in the carotid body and its clinical implications.

## Expression of RAS components in the carotid body

The expression and localization of several key RAS components, notably angiotensinogen, which is an indispensable component for the existence of an intrinsic RAS, have been detected in the rat carotid body (Lam and Leung, 2002). Hence, the mRNA and protein of angiotensinogen are expressed in the chemosensitive glomus cells. In addition, mRNA expression of ACE is present despite an absence of the expression of renin in the rat carotid body. These strongly support the localization of an intrinsic angiotensin-generating system functioning via a locally renin-independent biosynthetic pathway in the carotid body (Figure 1). In this regard, the local angiotensin generation of Ang I and Ang II could be mediated by renin in the circulating blood or functional homologous enzymes of renin including tonin, cathepsin G, and kallikrein (Figure 1). Also, angiotensin peptides could be generated by renin-like enzymes expressed in the vasculature (Campbell, 2003). However, the expression of these alternative enzymes in the carotid body has not been investigated. Nevertheness, Ang II locally produced in the carotid body could elevate the local Ang II levels functioning as a paracrine-autocrine signal via the activation of AT_1_ receptors expressed in the glomus cells.

## Expression and function of angiotensin receptors in the carotid body

Peripheral infusion of Ang II stimulates respiration in anesthetized animals (Potter and McCloskey, 1979; Ohtake and Jennings, 1993; Ohtake et al., 1993). The respiratory response to Ang II is in part mediated by the carotid chemoreflex because Ang II increases afferent activities of the carotid body (McQueen, 1981; Allen, 1998). The effect of Ang II may be mediated by the sympathetic and parasympathetic efferent fibers innervating the carotid body, which could increase the release of norepinephrine and the afferent activity of the carotid body (McQueen, 1981; Gonzalez et al., 1994). Nevertheless, evidence suggests that Ang II exerts its effect on the chemosensory component of the carotid body. Hence, Ang II at concentrations ranging from physiological to pharmacological levels induces a brief inhibition followed by an excitation of afferent carotid sinus nerve activity in the rat superfused carotid body preparation (Allen, 1998; Leung et al., 2000). The resting activity of the carotid sinus nerve is dose-dependently increased by about two folds upon a threshold concentration of 100 pM at the physiological level of Ang II under normoxic conditions (Allen, 1998; Leung et al., 2000). Also, both the inhibitory and excitatory effects of Ang II are blocked by losartan, supporting the ligand binding is mediated by the AT_1_ receptors. In addition, in an autoradiographic study, AT receptor-ligand bindings in the carotid body is not reduced by sympathetic nor afferent denervation of the carotid body (Allen, 1998). Indeed, Ang II elevates the level of intracellular calcium in the chemosensitive glomus cells (Fung et al., 2001). The intracellular calcium response to Ang II is blocked by losartan but not by an antagonist for AT_2_ receptors PD-123319, suggesting an involvement of AT_1_ receptors. Furthermore, AT_1_-immunostaining is positively localized in the lobule of the rat carotid body, strongly supporting the expression of AT_1_ receptors in the glomus cells clustering in glomeruli (Fung et al., 2001). In fact, AT_1_ receptors are co-localized with cells containing tyrosine hydroxylase, which is a cellular marker of the biosynthesis of catecholamines for the chemotransduction in the carotid body (Fung et al., 2002). However, the AT_1_-immunoreactivity is not found in all lobules of the parenchyma, meaning that the expression of AT_1_ receptors is not ubiquitous in the carotid body under physiological conditions (Fung et al., 2001). This is in consistent with functional data showing that the proportional amount of glomus cells responsive to Ang II is about 40% (Fung et al., 2001). Nevertheless, these findings are conclusive that AT_1_ receptors expressed in the chemosensitive glomus cell mediate the carotid chemoreceptor response to Ang II. It is known that Ang II binding of the AT_1_ receptor stimulates the phospholipase C pathway in the plasma membrane and leads to the formation of 1,2-diacylglycerol and inositol-1,4,5-triphosphate (IP_3_), which mobilizes the endoplasmic calcium to store and elevate intracellular calcium (Balla et al., 1998). It is speculated that similar intracellular pathways could mediate the effect of Ang II on the glomus cells and the details of the intracellular signaling pathways need further study.

The mRNA transcript of two subtypes of AT_1_ receptors, AT_1a_ and AT_1b_, is expressed in the rat carotid body (Leung et al., 2000; Fung et al., 2002). It has been shown that the AT_1*a*_ receptor is the main one accounting for the Ang II effect on the chemoreceptor, whereas AT_1b_ might have limited involvement, if any, in the early stage of the maturation. In the postnatal rat, there is an increase in the expression of AT_1a_ subtype but a decrease in the AT_1b_ subtype regulated by hypoxia (Fung et al., 2002). This suggests that the AT_1a_ receptor is the major subtype accounting for the Ang II effect on the carotid body (Fung et al., 2002). In addition to AT_1_ receptors, mRNA transcripts of the AT_2_ receptor were found in the carotid body (Leung et al., 2000; Fung et al., 2002). Activation of the AT_2_ receptor has a wide spectrum of effects for instances on vasodilation, apoptosis and anti-proliferation depending on the cell type (Padia and Carey, 2013). Although the AT_1_ receptor is the major one mediating the excitatory response of the carotid chemoreceptor, it is possible that Ang II can also exert its effects via the AT_2_ receptors in the carotid body.

It has been reported that AT_4_ and Mas receptors are expressed in the carotid body (Fung et al., 2007; Schultz, 2011). Ang IV is an Ang II metabolite containing the 3–8 fragment of the octapeptide, which exerts its physiological effect via the AT_4_ receptor. It has been reported that activation of AT_4_ receptors by Ang IV augments the release of acetylcholine in the hippocampus (Chai et al., 2004). In the rat carotid body, positive immunoreactivity against AT_4_ receptors was found in the chemosensitive glomus cells containing the tyrosine hydroxylase (Fung et al., 2007). It is speculated that AT_4_ receptors binding with Ang IV could enhance the excitatory effect of Ang II on the carotid chemoreceptor mediated by the AT_1_ receptor. Supporting this idea, the expression of AT_4_ receptors in the carotid body is significantly increased under chronically hypoxic condition. Also, Ang IV elevates the intracellular calcium level of the chemosensitive glomus cells despite at a high concentration (10 μM) of Ang IV (Fung et al., 2007). Thus, the AT_4_ receptor could be a signaling pathway of the RAS in parallel and/or complementary to the AT_1_ receptor activation. The physiological or pathophysiological significance, if any, of the AT_4_ receptor expressed in the carotid body has yet to be fully elucidated in future studies. As for the Mas receptor, it is a G-protein coupling receptor with high affinity binding with Ang (1-7), a biologically active peptide converted from Ang I and Ang II, respectively, by ACE2 and ACE. Recent study reported the expression of Mas receptors in the rabbit carotid body and also its decreased expression under a disease condition associated with heart failure (Schultz, 2011). The signaling cascade of Mas receptor activation has been known to induce vasodilation, which is possibly a negative modulation of the functional effects of AT_1_ receptors. Thus, the Mas receptor may be functionally important in the modulation of the RAS activity in the carotid body and its alteration under disease condtions could be pathologically significant as it may contribute to the imbalance of excitatory and inhibitory modulation of the carotid chemoreceptor activity in disease (Schultz, 2011).

## Functions of local RAS components in the carotid body

The circulating RAS is responsive to alterations in extracellular fluid volume, osmolarity, blood volume or sodium depletion, resulting in an elevated level of Ang II in the plasma (Reid et al., 1978; Matsusaka and Ichikawa, 1997). In addition to the vasoconstrictive effect of Ang II and its stimulating effect on the aldosterone secretion by the adrenal cortex, Ang II stimulates carotid chemoreceptors, which elicits the chemoreflex for the adjustment in cardiopulmonary and autonomic activities. Specifically, activation of the chemoreflex pathway is known to elevate renal sympathetic activities leading to the secretion of renin from the juxtaglomerular cells in the kidney, which could then increase sodium reabsorption and water intake (Honig, 1989; Marshall, 1994). Thus, the physiological significance of the effect of Ang II on the carotid chemoreceptor is that the baseline activity of the carotid chemoreceptor is regulated by Ang II in the circulating blood and also produced by the local RAS, which could elicit the chemoreflex in the absence of hypoxia, hypercapnia, or acidosis. As such, the carotid chemoreceptor could serve as an effector of Ang II for the regulation of blood pressure, electrolyte, and fluid homestasis via the chemoreflex pathway. Moreover, Ang II could potentiate the carotid chemoreceptor response to hypoxia. The plasma Ang II level significantly increases under hypoxic conditions (Zakheim et al., 1976). Also, the presence of local RAS in the carotid body could elevate the level of Ang II and also its metabolites in the local tissue, which could be a major source of Ang II for a more prominent effect on the modulation of the carotid chemoreceptor activity under hypoxic or disease conditions. Hence in parallel to the function of Ang II-sensitive neurons in the circumventricular organs of the brain (Ganong, 2000; McKinley et al., 2003), the carotid chemoreceptor can be an additional effector of Ang II, which confers and provides the peripheral signal integrating to the central output that alters sympathetic and parasympathetic activities for the regulation of cardiovascular and renal activities and also the electrolyte and fluid homeostasis under physiological, hypoxic, and disease conditions.

## Sustained hypoxia regulates RAS expression: implications on chronic obstructive pulmonary disease

It has been shown that proportion of Ang II-responsive glomus cells (*ca.* 80%) is increased by 2 folds in the carotid body of rats exposed to 10% inspired oxygen for 4 weeks (sustained hypoxia) (Leung et al., 2000; Fung et al., 2001, 2002). Also, elevated intracellular calcium levels induced by Ang II is three times higher following sustained hypoxia than the normoxic group, which is blocked by losartan (Leung et al., 2000; Fung et al., 2002). Importantly, AT_1_ receptor-mediated excitation of carotid body chemoreceptor activity is two times higher in rats exposed to sustained hypoxia than in controls (Leung et al., 2000; Fung et al., 2002). Thus, sustained hypoxia induces a significant increase in the sensitivity of the chemoreceptor response to Ang II. In fact, the mRNA expression of the AT_1_ and AT_2_ receptor is increased in the carotid body of adult rats exposed to sustained hypoxia (Leung et al., 2000). Also, there is an increased immunoreactivity of AT_1_receptors in glomic clusters containing tyrosine hydroxylase in the carotid body of rats with sustained hypoxia (Fung et al., 2002). Interestingly, in postnatal exposure to sustained hypoxia, the mRNA expression of AT_1a_ receptors but not the AT_1b_ subtype in the rat carotid body is upregulated, suggesting that the expression of AT_1_ receptor subtypes is differentially regulated by postnatal hypoxia (Fung et al., 2002). Thus, chronic hypoxemia is a major factor that increases the expression of AT_1a_ receptor at the transcriptional and protein level, resulting in a functional enchancement of the sensitivity of the carotid chemoreceptor to Ang II.

In addition to the upregulation of the AT_1_ receptor, sustained hypoxia induces increased expressions of several key components of the RAS in the rat carotid body. The effect of sustained hypoxia on the RAS component of the carotid body are: (i) it increases the mRNA and protein level of angiotensinogen expressed in the chemosensitive glomus cell, and (ii) elevated the mRNA expression and enzymatic activities of ACE (Lam and Leung, 2003; Lam et al., 2004). The significance of these RAS alterations regulated by hypoxia is that it could increase the local biosynthesis of Ang II and angiotensin peptides, which increases the carotid chemoreceptor activity; via the chemoreflex, changes the respiratory and cardiovascular activities to match metabolic needs and also adjustments of the autonomic and renal activities to regulate the electrolyte and fluid homeostasis in hypoxia (Honig, 1989). In addition, the increased expression of the AT_1_ receptor could raise the sensitivity of the carotid body to the electrolyte and fluid disturbance under hypoxic conditions. Indeed, the plasma concentration of Ang II rises in the first week of sustained hypoxia but it returns to a normoxic level by 2 weeks (Zakheim et al., 1976). The plasma renin activity has been reported to be unaltered or increased during hypoxia (Jain et al., 1990; Fletcher et al., 1999; Ip et al., 2002). Thus, the increased expression and activities of the RAS components could play a role in the augmented activity of the carotid chemoreceptor and the chemoreflex pathway by which increases and sustains the renal sympathetic activity under hypoxic conditions. This could lead to activation of the renin-angiotensin-aldosterone system, which is important to increase the sodium and water retention. This might be a part of the compensatory changes following the natriuretic and diuretic effects of carotid chemoreceptor stimulation in acute hypoxia (Honig, 1989). Consequently, the increased sensitivity of the carotid body to Ang II could play a role in the augmentation of cardiorespiratory and the renal sympathetic activities, which is an important part of the response to hypoxia. Moreover, Ang II levels are significantly elevated under pathological conditions for instances hypotension or hemorrhage. Indeed hypotension induces an increase in the discharge rate of carotid chemoreceptors which may be due to a decrease in the blood flow to the carotid body (Lahiri et al., 1980). The elevated carotid chemoreceptor activity could also be mediated by the AT_1_ receptors and the upregulated RAS components in the carotid body (Leung et al., 2000; Fung et al., 2002).

Sustained hypoxia is closely relevant to the physiological acclimation to high altitude, and also to clinical conditions including chronic obstructive pulmonary disease and congenital heart defects (Forth and Montgomery, 2003; Prabhakar and Peng, 2004). The upregulation of RAS components in the carotid body and the augmented sensitivity of the chemoreceptors to Ang II could play multiple roles in the response of the carotid body to sustained hypoxia. Specifically, the carotid body develops hypertrophy and hyperplasia and also increases vasculature with angiogenesis (Fung and Tipoe, 2003). Ang II is known as a mitogenic factor effecting on vascular cells, although the effect on the glomic tissue is not clear at the moment. In addition, sustained hypoxia modulates the ventilatory response to hypoxia (Bisgard, 2000; Lahiri et al., 2000, 2002). The chemosensitivity of the carotid body to hypoxia is modulated by the counterbalance of effects of excitatory and inhibitory components on the carotid chemoreceptor (Bisgard, 2000; Prabhakar, 2001). Thus, the excitatory effect of Ang II on the glomus cell could augment the chemosensitivity of the carotid chemoreceptor, which may counteract the blunting effect of sustained hypoxia on the ventilatory response to hypoxia. Yet, the extent of the effect of Ang II and the detail of the molecular and cellular mechanisms underlying the functional modulation of the chemoreceptor excitability for the acclimatized changes in the carotid body during hypoxia require further studies.

The RAS plays important roles in the pathogenesis of disease conditions including hypertension, cardiac hypertrophy and heart failure. It has been shown that AT_1_ receptor antagonist olmesartan significantly lowers the blood pressure and reduces proteinuria and glomerular damage mediated by oxidative stress in hypertensive diabetic animals (Izuhara et al., 2005). It has also been reported that ACE and Ang II play roles in the hypoxia-induced pulmonary hypertension and vascular remodeling (Morrell et al., 1995, 1999). The pathological development of hypoxic cor pulmonale is significantly decreased by olmesartan in rats exposed to sustained hypoxia (Nakamoto et al., 2005). However, in a double-blind study, losartan did not significantly attenuate pulmonary hypertension in a cohort of 40 patients with chronic obstructive pulmonary disease (Morrell et al., 2005). Also, irbesartan, an AT receptor blocker, did not significantly alter the strength of respiratory muscles or spirometric parameters in a randomized trial with about 60 patients with chronic obstructive pulmonary disease, although it significantly decreased the hematocrit. This raises the possibility that blockade of AT receptors may have beneficial effects in the patients with chronic obstructive pulmonary disease (Andreas et al., 2006). A more recent study reported that ACE inhibitor enalapril or AT receptor blocker losartan reduced the mortality of 2249 patients with severe COPD (Ekström et al., 2013). The effect of RAS blockade in disease associated with hypoxia needs more clinical trial studies in future.

## Intermittent hypoxia regulates RAS expression: implications on sleep-disordered breathing

Chronic exposure to episodic hypoxia (intermittent hypoxia) associated with recurrent apneas closely related to pathophysiological conditions including sleep-disordered breathing, obstructive sleep apnea and hypertension (Lesske et al., 1997; Fletcher, 2001). Evidence suggests that the carotid body plays a crucial role in the pathophysiology of sleep apnea and it pathophysiological consequences induced by intermittent hypoxia (Prabhakar et al., 2001, 2005; Peng et al., 2003; Iturriaga et al., 2005). Thus, there is an augmented carotid chemoreceptor activity and its chemosensitivity in animals exposed to intermittent hypoxia (Peng and Prabhakar, 2004; Peng et al., 2004; Rey et al., 2004). Also, intermittent hypoxia induces increases in the blood pressure (Fletcher et al., 1992a,b), activities of sympathetic nerve (Greenberg et al., 1999; Fletcher, 2003), plasm levels of catecholamines (Bao et al., 1997), long-term facilitation (LTF) of the respiratory motor activity and the ventilatory response to hypoxia (McGuire et al., 2004; Rey et al., 2004; Katayama et al., 2005). Moreover, denervation of the carotid afferent activity dramatically attenuates the elevated blood pressure in responding to intermittent hypoxia, indicating that the carotid chemoreceptor activity plays an important role in the pathogenic cascades induced by intermittent hypoxia (Fletcher et al., 1992a). Furthermore, intermittent hypoxia resembles ischemia-reperfusion of tissues and organs, leading to excessive production of ROS, which could underpin the long-term effects of intermittent hypoxia on the carotid body. This is supported by the observation that ROS scavengers attenuate the hypoxic sensitivity and the magnitude of LTF induced by intermittent hypoxia (Prabhakar and Kumar, 2004). Thus, ROS play a crucial role in the altered carotid body function in intermittent hypoxia.

As aforementioned, Ang II stimulates ventilation and the plasma Ang II level increases under hypoxic conditions (Zakheim et al., 1976; Ohtake et al., 1993). Activation of AT_1_ receptors in the carotid body increases the afferent activity and the hypoxic sensory response of the chemoreflex and sympathetic output (Leung et al., 2000). In experimental animals, losartan significantly reduces the elevated arterial pressure induced by intermittent hypoxia, suggesting that the RAS is involved in the pathogenic cascade (Fletcher et al., 1999). Indeed, there are significantly increased levels of serum Ang II and VEGF in patients with obstructive sleep apnea (Barcelo et al., 2001; Moller et al., 2003). Blocker of AT_1_ receptors olmesartan significantly decreases the VEGF expression induced by Ang II in the peripheral blood mononuclear cell (Takahashi et al., 2005). Thus, activation of the AT_1_ receptor plays a role in the pathogenic event of obstructive sleep apnea. Recent studies have examined the hypothesis that the RAS in the carotid body plays a role in the augmented carotid chemoreceptor activity induced by intermittent hypoxia, which may be mechanistically leading to breathing instability in the pathophysiology of sleep apnea.

Recent studies reported that intermittent hypoxia induces a functional upregulation of the RAS expression in the rat carotid body (Lam et al., 2014). The increased mRNA and protein expression of AT_1_ receptors causes an enhancement of the sensitivity to Ang II in the carotid chemosensitive cells, which could lead to an increase in the CB excitability and renal sympathetic activity (Marcus et al., 2010; Lam et al., 2014). In effect, the activation of RAS in the carotid body during intermittent hypoxia has pathophysiological and clinical significance because the chemoreflex plays an important role in the sustained increases in the sympathetic outflow and the systemic arterial pressure (Fletcher, 2001). Indeed, it has been shown that the systemic hypertension induced by intermittent hypoxia is blocked by the denervation of the carotid body, ablation of the sympathetic nerve, renal sympathectomy, adrenal medullectomy, and also by AT receptor antagonist (Fletcher et al., 1999; Fletcher, 2001). These data support the hypothesis that the RAS expression in the carotid body plays a role in the pathogenic cascade induced by intermittent hypoxia, which increases the cardiovascular morbidity in OSA patients.

Ang II stimulates the [Ca^2+^]_*i*_ elevation in the chemosensitive glomus cells and the Ang II response is enhanced in the hypoxic group via the upregulation of AT_1_ receptor expression (Lam et al., 2014). The [Ca^2+^]_*i*_ response to AT was inhibited by AT_1_ antagonist losartan but not by an AT_2_ antagonist, confirming that AT binding to the AT_1_ receptor stimulates the intracellular signaling pathway and elevates [Ca^2+^]_*i*_ in the glomus cells. In effect, Ang II evokes sensory long-term potentiation of the carotid body, which was blocked by losartan (Peng et al., 2011). Also, losartan reduced the elevated sympathetic responses to hypoxia and cyanide (Marcus et al., 2010). These results suggest that the upregulation of the AT_1_ receptor expression plays a role in the augmented CB excitability during intermittent hypoxia, highlighting the mechanistic significance of the RAS in mediating the IH-induced pathophysiological development of sympathetic overactivity and hypertension.

In addition to AT_1_ receptors, the gene transcripts of AT_2_ receptor have shown to be increased in the carotid body in intermittent hypoxia. The increased expression of the AT_2_ receptors might be related to the lack of increase in the volume of the carotid body in the rat (Lam et al., 2008). Indeed, AT_2_ receptors have been implicated in the stimulation of apoptosis and the activation of AT_2_ receptor results in growth inhibition and promotion of apoptosis associated with the inhibition of MAP kinases, such as extracellular regulated kinases, probably by the activation of phospho-tyrosine phosphatase (Schmitz and Berk, 1997; de Gasparo and Siragy, 1999). The activated local RAS via AT_2_ receptors in the carotid body might inhibit the cell growth and promote apoptotic cell death, resulting in an insignificant change in the volume of the carotid body in intermittent hypoxia. However, further investigations are needed for addressing the role of AT_2_ receptors in the carotid body.

Moreover, the expression of angiotensinogen in the carotid body is significantly elevated by intermittent hypoxia (Lam et al., 2014). The mRNA and protein expression of angiotensinogen was specifically localized to the glomus cells of the carotid body, suggesting a transcriptional upregulation and/or mRNA stabilization of the angiotensinogen expression induced by intermittent hypoxia. Also, the mRNA level of ACE was significantly increased in the rat carotid body (Lam et al., 2014). Thus, evidence supports that intermittent hypoxia induces an upregulation of the local RAS components in the rat carotid body. Since angiotensinogen is the sole precursor of Ang II, elevated levels of the expression of angiotensinogen could lead to an increase in the local production of Ang II in the carotid body in intermittent hypoxia. Moreover, the increased expression of ACE in the carotid body could enhance the kinetics of enzymatic convertion of Ang I to Ang II in the carotid body. Also, increased ACE activities and plasma Ang II levels have been reported in OSA patients (Barcelo et al., 2001; Moller et al., 2003). In effect, the elevated level of circulating and locally produced Ang II, together with an increased expression of the AT receptors could enhance the effect of Ang II on the chemosensory component of the carotid body. Thus, evidence supports the upregulated AT receptors with increased expression of RAS components in the carotid body play a pathogenic role in the augmented excitability of the carotid chemoreceptor under intermittent hypoxia associated with sleep apnea.

Oxidative stress with an increased generation of ROS plays an essential role in IH-induced alterations in the carotid body function (Prabhakar et al., 2001). It has been shown that ROS modulate the mobilization of [Ca^2+^]_*i*_ store mediated by IP_3_signaling pathway, leading to an increase in hypoxia-induced neurotransmitter release in IH (Prabhakar and Kumar, 2004). Studies have also shown that oxidative stress is involved in the augmented carotid chemoreceptor activity (Pawar et al., 2009; Peng et al., 2009; Del Rio et al., 2010, 2011b). Emerging data suggest that Ang II is a mediator of oxidative stress, in which ROS induced by Ang II are an important signaling intermediates in several signal transduction pathways involved in the pathophysiology (Paravicini and Touyz, 2006) and inflammation (Duprez, 2006). In this regard, vascular inflammation induced by Ang II is mainly mediated by AT_1_ receptors associated with an increased production of ROS via the NADPH oxidase in the vascular wall (Griendling et al., 1994; Rajagopalan et al., 1996; Dandona et al., 2003), which is closely related to the local RAS function (Shimizu et al., 1998). In the carotid body, AT receptors are also expressed in the vascular cells in addition to the glomus cells, although its role is unclear. Nevertheless, it has been shown that losartan treatment could normalize IH-induced superoxide production and expression of AT_1_ receptors (Marcus et al., 2010). Also, ROS production induced by Ang II mediates sensory long-term potentiation in the carotid body via activation of gp91^phox^ (Peng et al., 2011), and the gp91^phox^ is expressed in glomus cells of the CB (Youngson et al., 1997). Thus, the IH–induced RAS upregulation in the carotid body may contribute to the AT-induced ROS production. Furthermore, losartan treatment of the rat in intermittent hypoxia attenuates the levels of oxidative stress and macrophage infiltration, supporting a pathogenic role of AT_1_ receptors in the local inflammation of the carotid body (Lam et al., 2014). In this context, intermittent hypoxia induces increased expressions of proinflammatory cytokines and mediators as well as infiltration of immunogic cells to the carotid body under hypoxic condtions (Lam et al., 2008, 2012; Liu et al., 2009; Del Rio et al., 2011a, 2012). The inflammatory response of the carotid body to intermittent hypoxia has also been proposed to play a role in the augmented activity of the carotid chemoreceptor (Lam et al., 2008, 2012; Liu et al., 2009). These findings strongly suggest a paracrine-autocrine mechanism mediating altered functions of the carotid body, including the augmented chemosensitivity and the local inflammation of the carotid body. Thus, intermittent hypoxia could activate an intrinsic angiotensin-generating system, which increases local biosynthesis of Ang II via increased expressions of RAS components in the carotid body. The upregulation of angiotensinogen, AT receptors and ACE expression could play a pathogenic role in the augmented activity of carotid chemosensitive cells and the inflammation of the carotid body in intermittent hypoxia, which is relevant to the early pathogenesis in sleep-disordered breathing.

The RAS has been proposed to play a role in the cardiovascular consequences of sleep apnea, including systemic hypertension. In addition to the continuous positive airway pressure therapy, targeting the RAS has been proposed as a pharmacological management of the patients with sleep apnea. In fact, inhibitors of ACE have been shown to attenuate the arterial pressures and decrease the apnea and hypoapnea index in patients with obstructive sleep apnea (Peter et al., 1989; Mayer et al., 1990; Grote et al., 1995). Also, the AT receptor blockers have been shown to attenuate the elevated pressure and oxidative stress induced by intermittent hypoxia in subjects (Foster et al., 2010; Pialoux et al., 2011) or in the patient with obstructive sleep apnea (Heitmann et al., 2010; Dohi et al., 2011). The beneficial effect of the RAS blockade might be explained by diminished sympathetic and adrenergic activities (Fletcher, 2000, 2003). Nevertheless, these human studies were performed in a small number of patients and the long term treatment with RAS blockers has not been evaluated in clinical trials (Parati et al., 2013). Future clinical studies are required to support the antihypertensive benefit of blockade of RAS for the management of patients with obstructive sleep apnea and hypertension.

## An involvement of the RAS in heart failure

Experimental studies have shown that Ang II enhances the hypoxic chemosensitivity of the carotid body in a rabbit model of chronic heart failure (CHF). Hence, Ang II augments hypoxia-induced renal sympathetic nerve activity (RSNA) and there are significant increases in the expression of AT_1_ receptors in the carotid body of CHF rabbits (Li et al., 2006). Also, L-158809, an AT_1_ receptor antagonist, attenuates hypoxia-induced responses of the RSNA in CHF rabbits. In addition, L-158809 decreases the chemoreceptor responses to hypoxia in CHF rabbits, suggesting that increases in Ang II and the expression of AT_1_ receptor in the carotid body play a role in the augmented carotid chemoreceptor activity and chemoreflex-mediated sympathetic overactivity in CHF (Li et al., 2006). Furthermore, Ang II at a concentration of 0.1 nM increases the sensitivity of potassium (Kv) currents and resting membrane potential to hypoxia and L-158809 reduces the sensitivity of Kv currents and resting membrane potential to hypoxia in CHF glomus cells. These results suggest that Ang II-AT_1_ receptor signaling pathway increases the sensitivity of Kv channels to hypoxia in the glomus cells of the CHF rabbit (Li and Schultz, 2006). Moreover, the effect of Ang II on the augmented chemoreceptor activity is medated by a NADPH oxidase-superoxide signaling pathway (Li et al., 2007).

Sleep-disordered breathing with central or obstructive sleep apnea is frequently observed in patients with heart failure. Sleep-disordered breathing has been known to have a negative impact on the CHF patient and so clinical treatment of sleep-disordered breathing could improve cardiac performance and long-term outcomes in these patients. Also, cardiac dysfunction may play a role in the pathophysiology of sleep apnea, although the interrelationship between heart failure and sleep apnea remains to be established (Caples et al., 2005). In this context, an increase in sympathetic activities is a hallmark of the CHF state, which could be mediated by a decrease in the sensory feedback from cardiopulmonary activities and arterial baroreceptors. As mentioned above, the sustained increase in RSNA could involve Ang II and the RAS in the carotid body of CHF rabbits (Schultz, 2011; Patel and Schultz, 2013). Indeed, recent studies show that cryoablation of the carotid body normalizes the RSNA and breathing stability and improves survival in CHF animals (Del Rio et al., 2013; Marcus et al., 2014). Hence, the RAS components could be therapeutic targets for the treatment of CHF patients.

## Conclusions

Findings of expression and functional studies suggest that the AT_1_ receptor regulates the excitability of the carotid chemoreceptor. Hence, Ang II elevates the level of intracellular calcium in the chemosensitive glomus cells and the activity of carotid chemoreceptors. As a result, activation of the chemoreflex could be a peripheral control important for the physiological response to hypoxia and the maintenance of electrolyte and fluid homeostasis. In addition, the expression of AT receptors in the carotid body is regulated by hypoxia. In effect, sustained hypoxia induces an upregulation of AT_1_ receptor expression, which increases the sensitivity of the chemoreceptor response to Ang II. This regulation may be important in the modulation of the carotid body functions responsible for the hypoxic ventilatory response, for enhancing the cardiorespiratory response and adjusting electrolyte and water homeostasis during sustained hypoxia. Furthermore, RAS components are locally expressed in the carotid body and the increased RAS expressions are closely relevant to the pathogenesis of disease including sleep-disordered breathing and heart failure. Specifically, the upregulation of the expression of angiotensinogen, ACE and AT_1_ receptors could play a significant role in the augmented carotid chemoreceptor activity, via the increased activity of chemoreflex, contributing to the pathophysiology of sleep apnea and the sympatho-excitation that is central to the endothelial dysfunction and heart failure during the course of pathogenesis. Future studies in this direction warrant a better understanding of the pathogenic role of RAS in the carotid body in the disease associated with hypoxemia.

### Conflict of interest statement

The author declares that the research was conducted in the absence of any commercial or financial relationships that could be construed as a potential conflict of interest.
